# Change of Conduction Mechanism in Polymer/Single Wall Carbon Nanotube Composites upon Introduction of Ionic Liquids and Their Investigation by Transient Absorption Spectroscopy: Implication for Thermoelectric Applications

**DOI:** 10.1021/acsanm.3c01735

**Published:** 2023-07-07

**Authors:** Beate Krause, Ioannis Konidakis, Emmanuel Stratakis, Petra Pötschke

**Affiliations:** † Leibniz-Institut für Polymerforschung Dresden e.V. (IPF), Hohe Str. 6, 01069 Dresden, Germany; ‡ Foundation for Research and Technology-Hellas (FORTH), Institute of Electronic Structure and Laser (IESL), 70013 Heraklion-Crete, Greece

**Keywords:** carbon nanotube fillers, thermoelectric polymer composites, ionic liquids, exciton dynamics, free-charge carrier lifetimes, time-resolved transient absorption spectroscopy

## Abstract

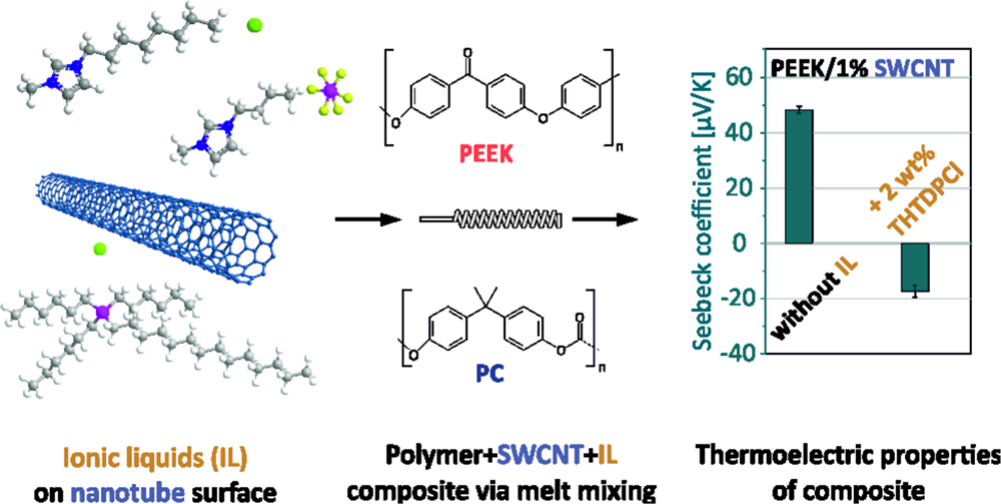

Polymer composites based on polycarbonate (PC) and polyether ether ketone (PEEK) filled with single-walled carbon nanotubes (SWCNTs, 0.5–2.0 wt %) were melt-mixed to investigate their suitability for thermoelectric applications. Both types of polymer composites exhibited positive Seebeck coefficients (*S*), indicative for p-type thermoelectric materials. As an additive to improve the thermoelectric performance, three different ionic liquids (ILs), specifically THTDPCl, BMIMPF6, and OMIMCl, were added with the aim to change the thermoelectric conduction type of the composites from p-type to n-type. It was found that in both composite types, among the three ILs employed, only the phosphonium-based IL THTDPCl was able to activate the p- to n-type switching. Moreover, it is revealed that for the thermoelectric parameters and performance, the SWCNT:lL ratio plays a role. In the selected systems, *S*-values between 61.3 μV/K (PEEK/0.75 wt % SWCNT) and −37.1 μV/K (PEEK/0.75 wt % SWCNT + 3 wt % THTDPCl) were reached. In order to shed light on the physical origins of the thermoelectric properties, the PC-based composites were studied using ultrafast laser time-resolved transient absorption spectroscopy (TAS). The TAS studies revealed that the introduction of ILs in the developed PC/CNT composites leads to the formation of biexcitons when compared to the IL-free composites. Moreover, no direct correlation between S and exciton lifetimes was found for the IL-containing composites. Instead, the exciton lifetime decreases while the conductivity seems to increase due to the availability of more free-charge carriers in the polymer matrix.

## 1 Introduction

In context with the continuous need of more efficient use of energy and more sustainable energy sources, thermoelectric (TE) based energy conversion is increasingly important. Using TE materials, the generation of a thermovoltage is possible through a temperature gradient on both sides of such materials allowing, e.g., to harvest waste heat. To characterize the TE properties of a material, the Seebeck coefficient (*S*) is used which represents the quotient between the generated thermovoltage (Δ*V*) and the applied temperature difference (Δ*T*). Considering also the electrical conductivity (σ) of a sample, the power factor (PF) can be calculated by multiplying the squared *S* with σ. In addition, the figure of merit (ZT) can be obtained by also taking into consideration the thermal conductivity κ of a sample by the formula ZT = PF·*T*/κ. For the generation of advanced TE modules, the combination of materials with p-type conduction behavior (positive *S* value) and n-type behavior (negative *S* value) is favorable and typically used.

The material groups typically used for TE applications are half-Heusler-compounds, clathrates, silicides, antimonides, and tellurides.^1−11^ In the recent past, a large number of publications have been published on the TE properties of carbon nanotubes (CNTs) themselves and CNTs modified with additives,^12−21^ as well as on intrinsically conductive polymers (ICPs).^10,17,22−25^

In the last few years, polymer-based materials have become increasingly important as they represent much more environmentally friendly solutions. In addition, they are much cheaper, easier to produce and process, and have intrinsically low thermal conductivity. Next to ICPs and their composites, in the last years, electrically conductive polymer nanocomposites (CPCs) based on melt-processable insulating polymers combined with electrically conductive (nano) fillers came more intensively in the research focus. Especially fillers with high aspect ratios, such as CNTs form electrically conductive networks in a polymer matrix already at low loadings (0.1–5 wt %).^26^ Examples from the literature of the use of commercial CNTs to prepare melt-mixed composites are shown for the thermoplastic matrices polypropylene (PP), polycarbonate (PC), poly(ether ether ketone) (PEEK), polyvinylidene fluoride PVDF, and poly(butylene terephthalate) (PBT).^27−36^ Such composites typically exhibit p-type conduction character with positive *S* values. However, even when using p-type CNTs, n-type conduction behavior (negative *S* values) was found for certain CNTs in certain matrices. Such polymers are acrylonitrile butadiene styrene (ABS) and different kinds of polyamides (PAs). It is assumed that these polymers act as electron donators to the nanotubes, thus changing the character of the composites to negative *S* values.^32^ In addition, it is possible to add n-type CNTs, such as nitrogen-doped ones, to polymers to obtain n-type composites.^37^ Another way shown before in the literature, is to combine CNTs with certain doping additives before they are incorporated in the polymer matrix by melt mixing.^21,38^ The use of PEG,^28,39^ PEI,^40^ as well as PVP^41^ was reported in the literature.

In this study, another type of additive, namely, ionic liquids (ILs) are used with the aim to modify intrinsically p-type single-walled CNTs (SWCNTs) into n-type melt-mixed composites based on the matrix polymers PC and PEEK. The two polymers were already used in a former study^33^ in which the effect of different kinds of CNTs and their combinations in hybrid filler systems on the TE properties of their composites was studied. These selected polymer matrices were used to test whether the effects of the CNTs on the TE properties depend on the type of polymer matrix, whereby PC represents an amorphous and PEEK represents a partially crystalline polymer and all composites had p-type behavior. ILs were described as modifiers for CNTs^42^ and polymers^43,44^ to change a wide range of properties. There are already some reports about TE properties when using ILs as an additive in melt-mixed composites with CNTs. Typically, ILs improve the dispersion of the fillers and thus, higher electrical conductivities can be achieved or the electrical percolation threshold can be reduced.^44−54^ For PP/single-walled CNT (SWCNT)-based composites, Luo et al. showed that the addition of the IL OMIM-BF_4_ resulted in a significant increase in electrical conductivity, as well as Seebeck coefficient, however, the conduction behavior remained p-type.^55^ Voigt et al.^56^ compared the effect of five kinds of ILs on the TE properties of PP/SWCNT composites and found that switching from p- to n-type behavior is possible depending on the IL structure. In their case, four of the five ILs were able to switch the conduction behavior.

Meanwhile, the exciton dynamics in CNTs and CNT-containing polymer composites have been the subject of several studies over the last decade.^33,37,57−61^ In particular, the employment of ultrafast laser time-resolved transient absorption spectroscopy (TAS) offers an excellent tool for studying the exciton lifetimes and recombination of picosecond regime processes, that are known to strongly determine the optical and electronic characteristics of the CNT fillers. Namely, the operation principle of TAS relies on a light source that is used to photoexcite electrons, while the corresponding decay dynamics of the relaxation processes are monitored in terms of optical absorption within various time delays.^33,37^ It has been shown that in SWCNTs, which are an excellent approximation of one-dimensional quantum confinement, free charge carriers instantaneously develop upon photoexcitation within several picoseconds,^59^ while electron excitations to higher energy states with CNTs are also plausible.^60^ Specifically, in energy harvesting and converting devices, such as perovskite solar cells^62^ and TE materials,^33,37^ the correlation between the obtained exciton dynamics and the power conversion efficiency is striking. Indeed, it was shown that longer exciton lifetimes and slower recombination rates are indicative of improved performance and stability.^33,37,62^ More specifically, it was found that in polymer-based composites with polycarbonate (PC) as the matrix and CNTs as fillers, the Seebeck coefficient exhibits a direct correlation with the exciton lifetimes, while being nearly independent of the CNT concentration within the host polymer matrix.^33^ This relationship between *S* and the exciton lifetime applies to both single CNT fillers and systems with combinations of two different CNT fillers (hybrid fillers).

In the present study, three different ILs in varied amounts were applied in PC and PEEK based composites with SWCNTs of different concentrations and the effect of the dopant on electrical conductivity and sign and value of the Seebeck coefficient, as well as morphology is thoroughly explored. The materials and preparation steps are illustrated in Schema 1. Moreover, TAS was employed in order to elucidate the effect of the addition on the conduction mechanisms in the developed PC/CNTs + IL composites. In addition to previous studies, the results reveal important details on the physical origins of the conduction mechanism and charge carrier processes within the IL containing PC-based polymer composites.

**Scheme 1:**
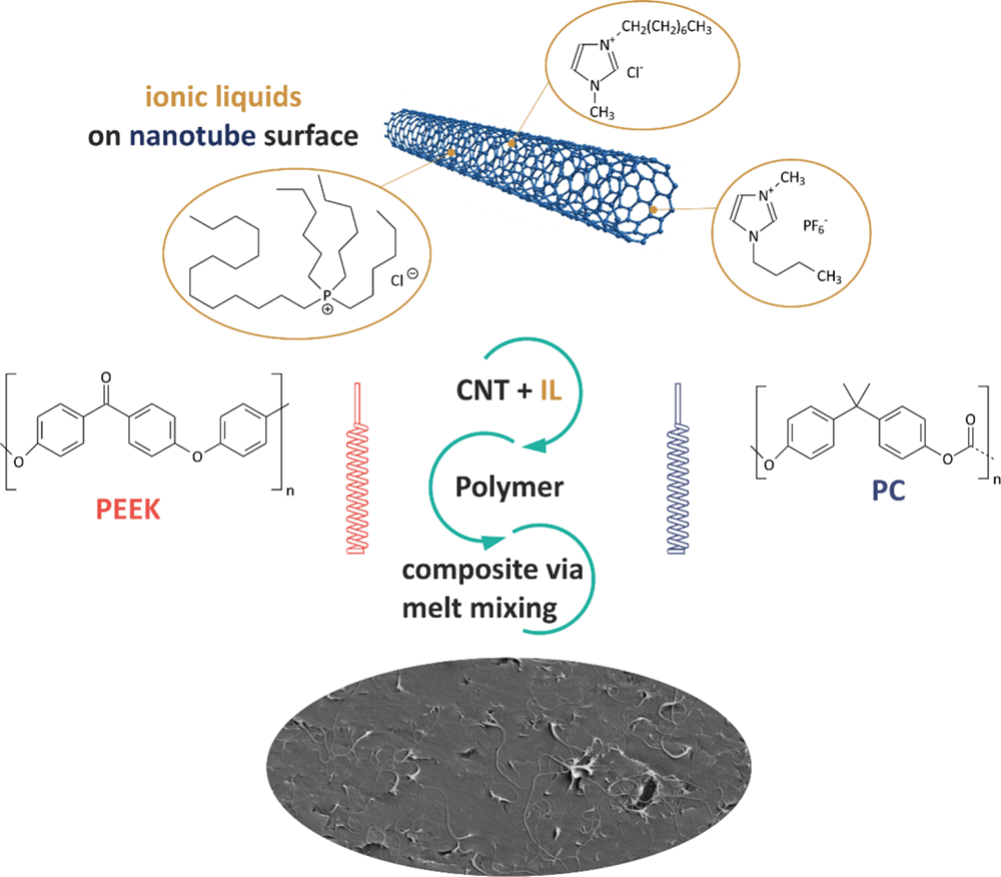
Chemical Structure of All Materials Used in This Study and Nanocomposite Preparation Way

## 2 Experimental Part

### 2.1 Material

As polymer matrices polycarbonate (Makrolon 2600, Bayer MaterialScience, Germany, abbreviation PC) with a density of 1.2 g/cm^3^ and a melt volume-flow rate (MVR) of 12 cm^3^/10 min (300 °C/1.2 kg), as well as polyether ether ketone (Vestakeep 1000P, Evonik, Germany, abbreviation PEEK), a material with a density of 1.3 g/cm^3^ and a MVR of 140 °C (380 °C/5 kg), were selected. As an electrically conductive filler, SWCNTs of the type Tuball (carbon purity 75%, OCSiAl S.a.r.l., Luxembourg, Luxembourg, abbreviation SWCNT) were chosen for this study.^63^

Several ILs were investigated as additives for the polymer/SWCNT composites. The IL selection is based on the results of Voigt et al.^56^ The used ILs are in particular 1-methyl-3-octyl-imidazolium chloride (OMIMCl; CAS 64697-40-1, conductivity @ 25 °C 0.09 mS/cm, purity > 97%, HPLC, Sigma Aldrich), 1-butyl-3-methyl-imidazolium hexafluoro-phosphate (BMIMPF6; CAS 174501-64-5, conductivity @ 25 °C 1.92 mS/cm, purity > 97.0%, HPLC, Sigma Aldrich), and trihexyltetradecylphosphonium chloride (THTDPCI; CAS 258864-54-9, conductivity @ 25 °C 4.63 mS/cm, IoLiTec-Ionic Liquids Technologies GmbH, Germany). All ILs are liquid at room temperature. The ILs differ in their polarity. OMIMCl is soluble in polar solvents like water or acetone and not in toluene and hexane. BMIMPF6 is only miscible with acetone. THTDPCl has more a non-polar character as it is soluble in non-polar solvents like toluene and hexane. The thermogravimetric analysis of ILs in air was determined (Figure S1).

### 2.2 Methods

Melt mixing of the composites was performed in a small-scale conical twin-screw micro compounder (Xplore Instruments BV, Sittard, The Netherlands) having a volume of 15 cm^3^. The composites were prepared at 280 °C (PC) or 360 °C (PEEK) with a rotation speed of 250 rpm for 5 min. These conditions were selected based on previous investigations.^33^ Before introducing in the compounder, the corresponding amount of IL was dropped on the SWCNT powder and mixed using a pestle and mortar. The polymer granules (PC) or polymer powder (PEEK) and CNT/IL premixture were alternately filled into the main hopper of the compounder. The extruded strands were compressing molded at the melt mixing temperature for 1 min into plates (60 mm diameter, 0.3 mm or 10 μm thickness) using the hot press PW40EH (Paul-Otto Weber GmbH, Remshalden, Germany). Strips cut from such plates were used as films for the measurements of the TE properties. All CNT contents are given in wt% based on the polymer weight.

The morphological characterization of the composites was performed using scanning electron microscopy (SEM) by means of a Carl Zeiss Ultra plus microscope combined with an SE2 detector. Before imaging, the composite strands were cryo-fractured in liquid nitrogen, and the surfaces were coated with 3 nm platinum.

The TE measurements were performed with the home-built facility at IPF at 40 °C with temperature differences between the two copper electrodes of up to 8 K (8 steps per 2 K).^64^ The ends of the samples (thickness 0.3 mm) were painted with conductive silver to reduce the contact resistances. The electrical resistivity was measured using a 4-point-arrangement. A Keithley multimeter DMM2001 was used to measure the thermovoltage, as well as the electrical resistance. All reported values represent mean values of 3–5 measurements on two strips.

Time-resolvedTAS measurements on the PC-based samples were performed on a Newport (TAS-1) transient absorption spectrometer, which is depicted schematically in Figure S4 in Konidakis et al.,^33^ while explained in detail elsewhere.^37,62^ The employed light source was a Yb:KGW pulsed laser with a central wavelength at 1026 nm, a pulse duration of 170 fs, and a repetition rate of 1 kHz. Such excitation parameters induce among other two-photon absorption processes. The detection range was set between 550 and 910 nm, while a pump fluence of 15 mJ/cm^2^ was used. The TE polymer composite samples were studied in the shape of compression-molded thin films with a thickness of 10 μm at room temperature. Several spots per sample were tested having similar signals (transmitted light). Areas with considerably less light transmission, indicating possible agglomerates, were avoided. Notably, PEEK-based composite films could not be studied by TAS due to missing translucency. TAS measurements of the OMIMCl IL were obtained upon employing identical excitation conditions while placing the liquid sample within a transparent cuvette. All TAS measurements were performed at room temperature. The measured difference in optical density (ΔOD) in the employed TAS spectrometer has been set us ΔOD = log(blocked/unblocked) = log(blocked) – log(unblocked) = OD_probe_ – OD_pump+probe._^33^ The error of the exciton lifetime determinations is ±0.1 ps.

## 3 Results and Discussion

### 3.1 Thermoelectric Performance

**Figure 1:**
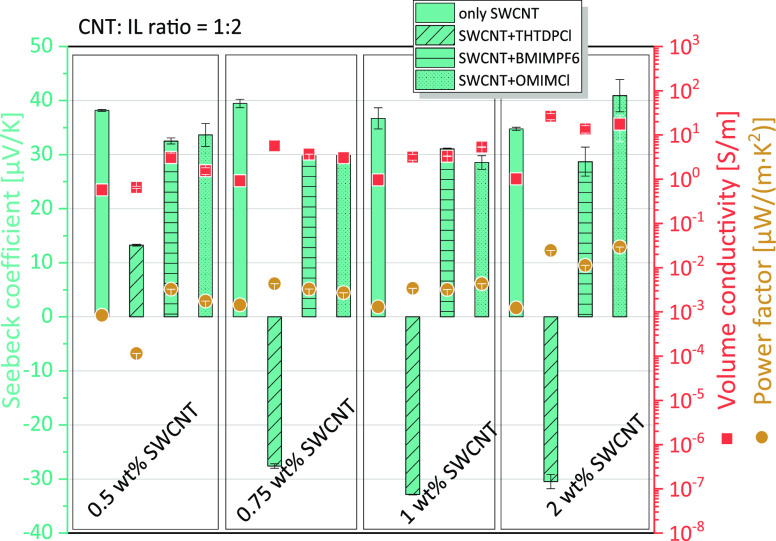
TE performance of PC composites with 0.5 to 2 wt % SWCNT, without and with different amounts of ILs, CNT:IL ratio 1:2.

As a screening step, different ILs were incorporated into PC composites at 0.5, 0.75, 1, and 2 wt % SWCNT, using a CNT:IL ratio of 1:2 (Figure 1). Especially for the IL THTDPCl, a significant influence on the TE behavior was found, as for the PC filled with 0.5–1 wt % SWCNT composites, the Seebeck coefficient was significantly reduced from 36.7–39.5 μV/K to 11.0–17.9 μV/K. For the PC/2 wt % SWCNT composite, the sign changed from positive to negative with −30.5 μV/K. For the other two ILs BMIMPF6 and OMIMCl, only a slight reduction of the Seebeck coefficient to 32.5–33.6 μV/K (at 0.5 wt % SWCNT), to 30.0–30.8 μV/K (at 0.75 wt % SWCNT), and 30.8 and 28.2 μV/K (at 1 wt % SWCNT), respectively, was measured. For the BMIMPF6 addition in PC/2 wt % SWCNT composite, also a slightly reduction of *S* value to 28.7 μV/K instead of 37.8 μV/K (without IL) was observed. For the PC/2 wt % SWCNT + 4 wt % OMIMCl composite, no significant change was found, possibly due to the large inhomogeneity of this composite, which is reflected in a high standard variation of the *S* value of ±3 μV/K. It is worth mentioning that the electrical conductivity for all composites with IL is higher than without IL, which is due to the intrinsic conductivity of the ILs.

**Figure 2:**
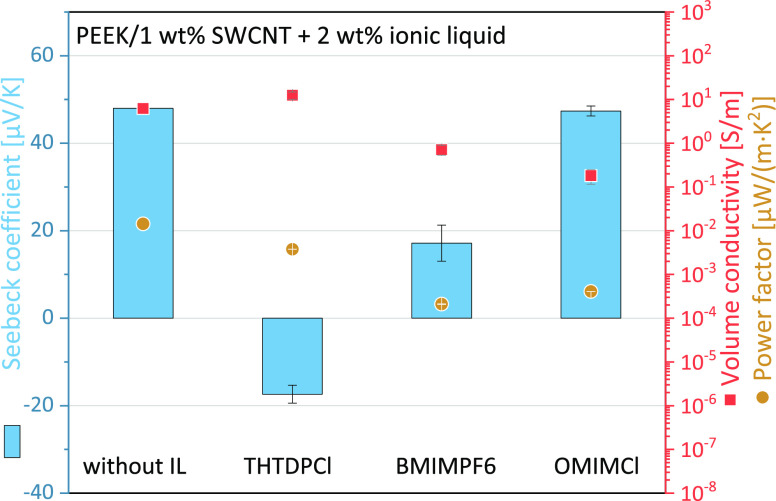
TE performance of PEEK/1 wt % Tuball composites without and with 2 wt % Ionic liquids (SWCNT:IL ratio 1:2).

A comparable influence of IL on the TE behavior of the composites was also determined for PEEK/1 wt % SWCNT composites with the same SWCNT:IL ratio of 1:2 (Figure 2 and Table S3). Only the addition of THTDPCl leads to a change to a negative Seebeck coefficient of −17.4 μV/K. For the other two ILs, a reduction of the *S* value from 53.0 to 15.8 μV/K (BMIMPF6) and 39.8 μV/K (OMIMCl) was measured. One reason why a reduction of the Seebeck coefficient is achieved, especially with the phosphonium-based IL, could be the high processing condition of 360 °C. Under a nitrogen atmosphere, the thermal degradation of the imidazole-based ILs starts around 250 °C (OMIMCl)^65^ and 373 °C (BMIMPF6),^66^ but for phosphonium-based IL, only at over 400 °C.^67^ Thus, there seems to be a correlation between the change in the TE behavior of the composites and the thermal degradation of the ILs. Furthermore, it should be taken into account that no inert atmosphere was present during the mixing of the melt. Even if it can be assumed that air exclusion is given after mixing the SWCNT and IL into the polymer melt, thermal degradation will have started. In the air, thermal degradation for the ILs starts at slightly lower temperatures (see Figure S1).

**Figure 3:**
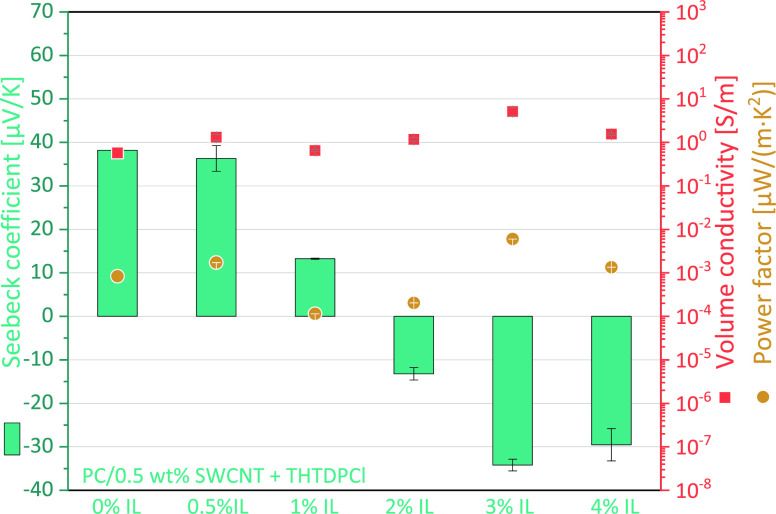
TE performance of PC/0.5 wt % SWCNT composites without and with different amounts of IL THTDPCls.

**Figure 4:**
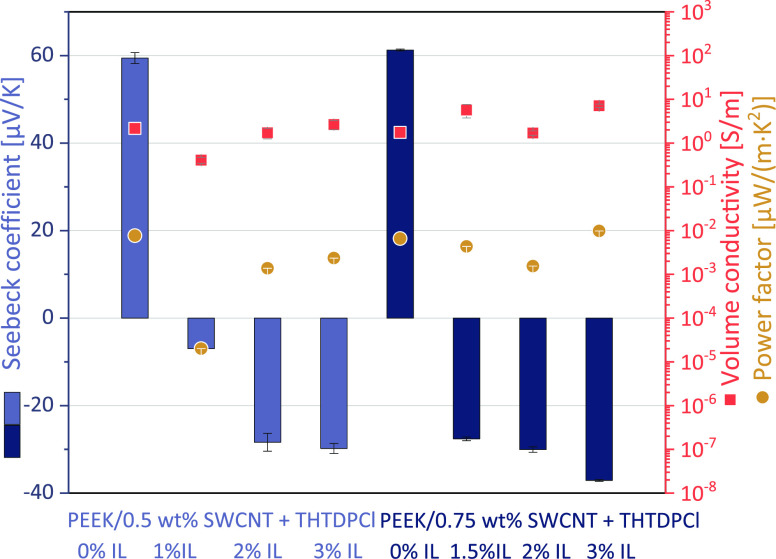
TE performance of PEEK/SWCNT composites (0.5–0.75 wt % SWCNT) without and with different amounts of ionic liquid THTDPCl.

Due to the aim of developing composite materials with a negative Seebeck coefficient, further tests were only carried out with IL THTDPCl. Furthermore, it was investigated whether the SWCNT:IL ratio or the total IL content is important to achieve a high negative S value. For this purpose, different SWCNT:IL ratios were incorporated into PC (Figure 3) and PEEK (Figure 4 and Table S4). For PC/0.5 wt SWCNT composites, it was found that an increasing amount of IL THTDPCl leads to a decrease in the positive Seebeck coefficient and at higher IL content to negative *S* values (Figure 3). It seems that an optimal content was reached at 3 wt % THTDPCl, since the *S* value was most negative here. The difference in the TE performance between 3 and 4 wt % IL was relatively small, whereby the high uncertainty of the *S* value at 4 wt % IL addition must be considered.

The PEEK composites also showed that the *S* values became more and more negative with increasing IL content (Figure 4). It seems that at an IL concentration of 2–3 wt %, a plateau is already reached with regard to TE performance with about −30 μV/K. The similar *S* values at the same IL-concentration and different SWCNT content lead to the conclusion that the absolute IL-content is more decisive for the *S* value than the ratio of SWCNT to IL. It can be concluded that a certain minimum amount of IL must be present in the composite to achieve a significant reduction of the Seebeck coefficient. This was found both for the PC composites, where a high negative *S* value was achieved starting from 3 wt % IL, and for the PEEK composites, where more than 1 wt % IL should be present in the composite. It can be assumed that the differences in the minimum amount of IL between PC and PEEK composites to achieve high negative *S* values are due to the effect of crystallinity. In amorphous PC, the entire polymer volume is available to the fillers, whereas, in semi-crystalline PEEK, the fillers cannot be localized in the crystals themselves. Therefore, the actual filler concentration in the amorphous part of a semi-crystalline polymer is higher than in an amorphous polymer.

### 3.2 Morphological Characterization

**Figure 5:**
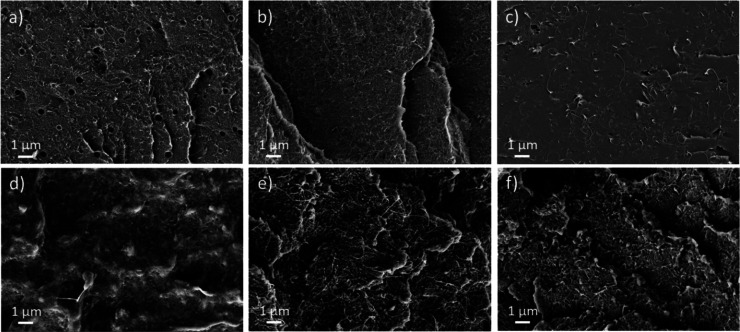
SEM of cryofractured surfaces of composites: (a) PC/2 wt % SWCNT + 4 wt % THTDPCl, (b) PC/2 wt % SWCNT + 4 wt % BMIMPF6, (c) PC/2 wt % SWCNT + 4 wt % OMIMCl, (d) PEEK/1 wt % SWCNT + 2 wt % THTDPCl, (e) PEEK/1 wt % SWCNT + 2 wt % BMIMPF6, (f) PEEK/1 wt % SWCNT + 2 wt % OMIMCl.

In a SEM study, the nanotube dispersion and the miscibility of the polymer matrix and IL additive were characterized. In case of immiscibility, the IL component is expected to be visible as droplets in the polymer matrix. On the cryofractured surfaces of all PC-based composites, the SWCNTs are visible as uniformly distributed light gray lines on the polymer surface (a–c). Only for the PC composite with THTDPCl were droplets visible, indicating immiscibility. This result is not surprising, as THTDPCl is a non-polar substance, unlike the other two ILs, which are more polar. Since polycarbonate is a more polar polymer, immiscibility with THTDPCl was to be expected. Figure 5d–f shows the SEM images of the PEEK/SWCNT composites, which also show a homogeneous CNT distribution. These images confirm the miscibility for all three IL types with the PEEK matrix. However, the surface of the PEEK composite containing THTDPCl appears somewhat blurred.

### 3.3 Transient Absorption Spectroscopy (TAS) and Exciton Dynamics

**Figure 6:**
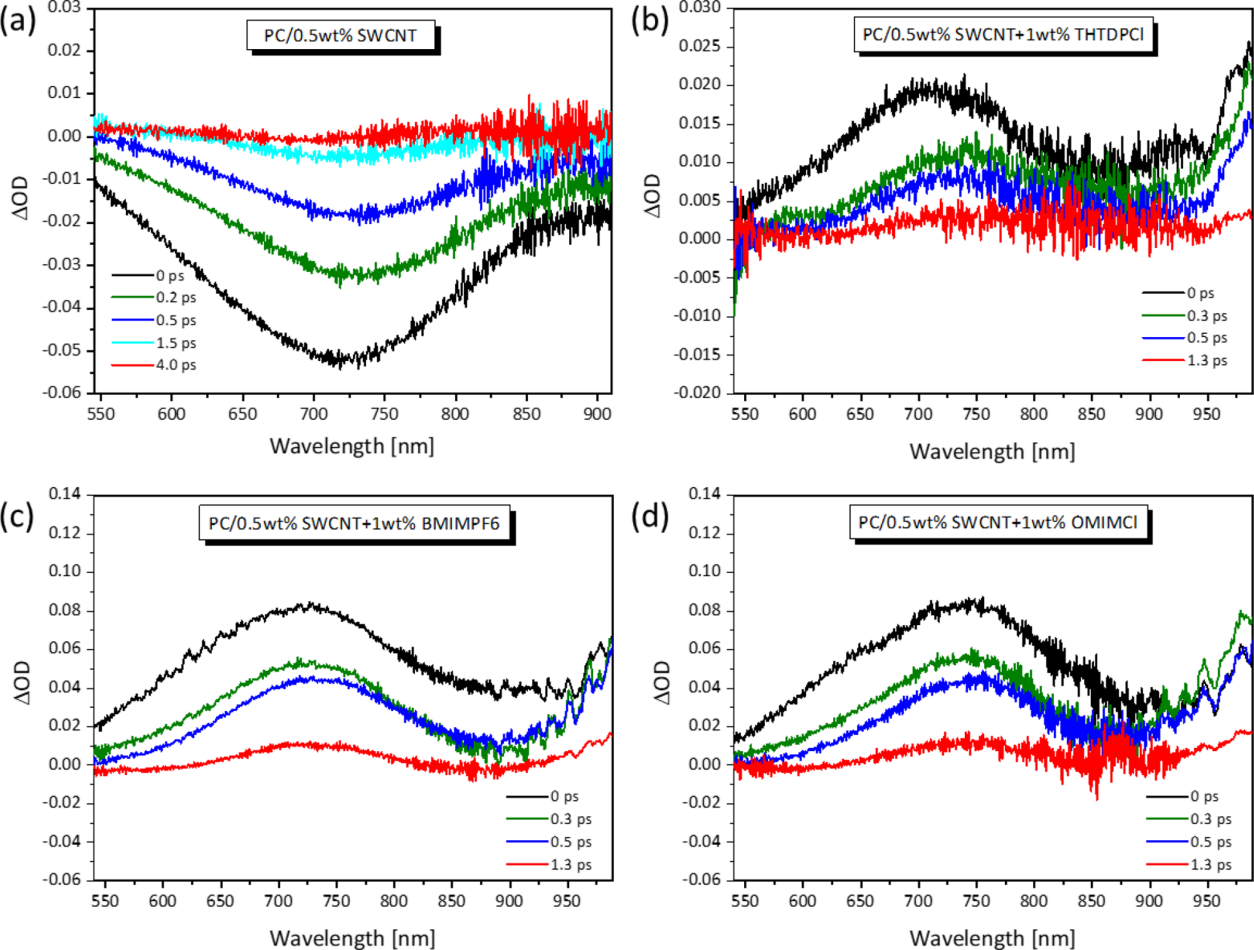
TAS spectra of the difference in optical density (ΔOD) as a function of wavelength for various delay times of (a) PC/0.5 wt % SWCNT and PC/0.5 wt % SWCNT + 1 wt % IL, where the used IL is (b) THTDPCl, (c) BMIMPF6, and (d) OMIMCl.

**Figure 7:**
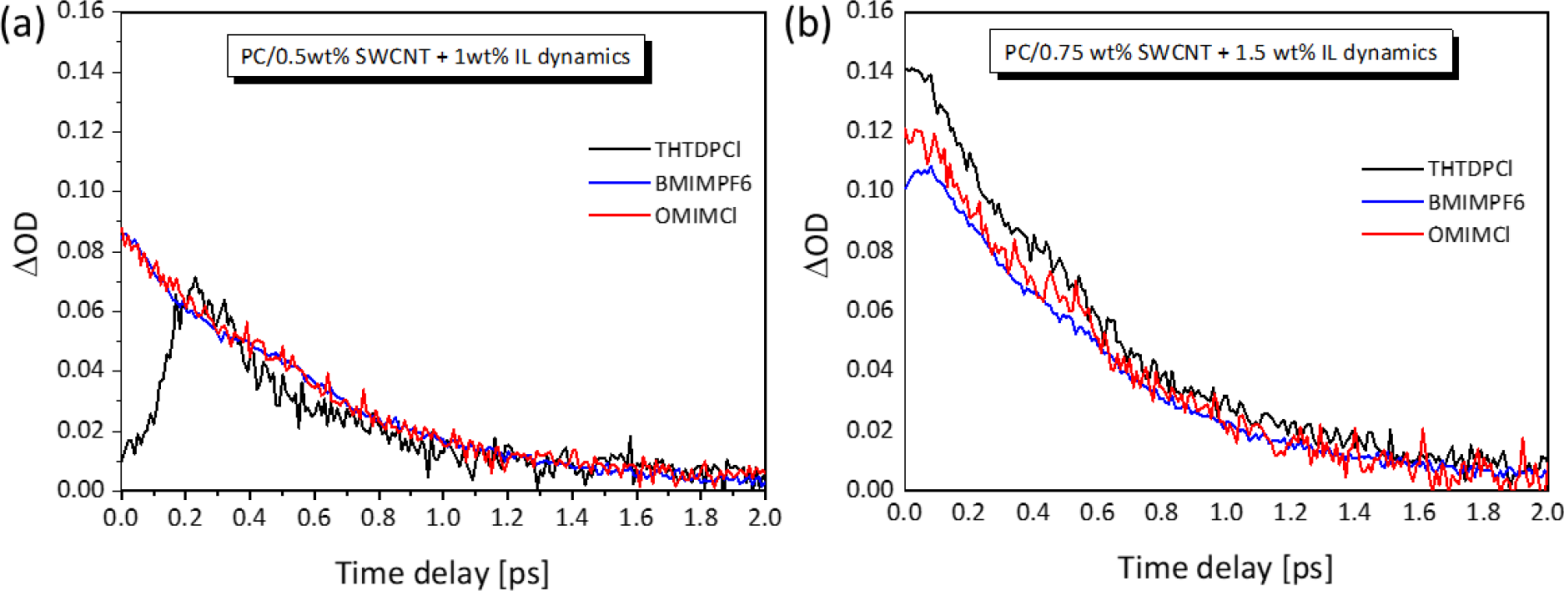
Exciton dynamics of (a) PC/0.5 wt % SWCNT + 1 wt % ILs and (b) PC/0.75 wt % SWCNT + 1.5 wt % ILs composites.

Targeting to explore further the effect of introducing ILs into the developed PC/SWCNT composites on their TE characteristics, the PC samples were examined by means of ultrafast laser time-resolved TAS. Figure 6a presents the TAS spectra of the difference in optical density (ΔOD) as a function of wavelength for various delay times of the PC/0.5 wt % SWCNT composite without IL addition and for the composites to which 1 wt % of IL was added, i.e., with a CNT/IL ratio of 1:2. Inspection of Figure 7a clearly shows that the PC/0.5 wt % SWCNT composite without IL addition exhibits negative optical density upon excitation, indicative of photobleaching (PB) behavior.^33,62^ In such conditions, the obtained photobleaching is attributed to the rapid relaxation of excitons from higher states to the E_11_ level.^57^ Notably, this process occurs within the laser pulse duration as it only requires around 100 fs.^57^ In particular, this finding was found to hold for all single-filler and hybrid-filler PC-based composites investigated in a previous study by TAS.^33^ On the contrary, the situation is different when the ILs are incorporated to the PC/0.5 wt % SWCNT composite. Inspection of Figure 6b–d reveals that when any of the three ILs is added to the PC/SWCNT composite, the resulting PC/0.5 wt % SWCNT + 1 wt % IL composites exhibit positive optical densities under the same photoexcitation conditions. Based on the paper by Gao et al.,^57^ the photo absorption (PA) band for SWCNTs at around 1.7 eV, i.e., equal to 729.3 nm, is attributed to transitions from E_11_ to E_33_, i.e., from E_11_ to the E_11_ + E_22_ manifold. Indeed, it is stated that the population at higher excitonic levels relaxes to the E11 subband within the laser pulse, and thus, only transitions from E_11_ exciton manifold can contribute to the obtained PA. Moreover, the authors attributed the red-shift of the PA band, also observed in this study, to the finite binding energy of the biexciton state.^57,62^ As depicted in Figure S2, the same optical density features hold for the PC/0.75 wt % SWCNT and PC/0.75 wt % SWCNT + 1.5 wt % IL composites with a CNT/IL ratio of 1:2.

**Figure 8:**
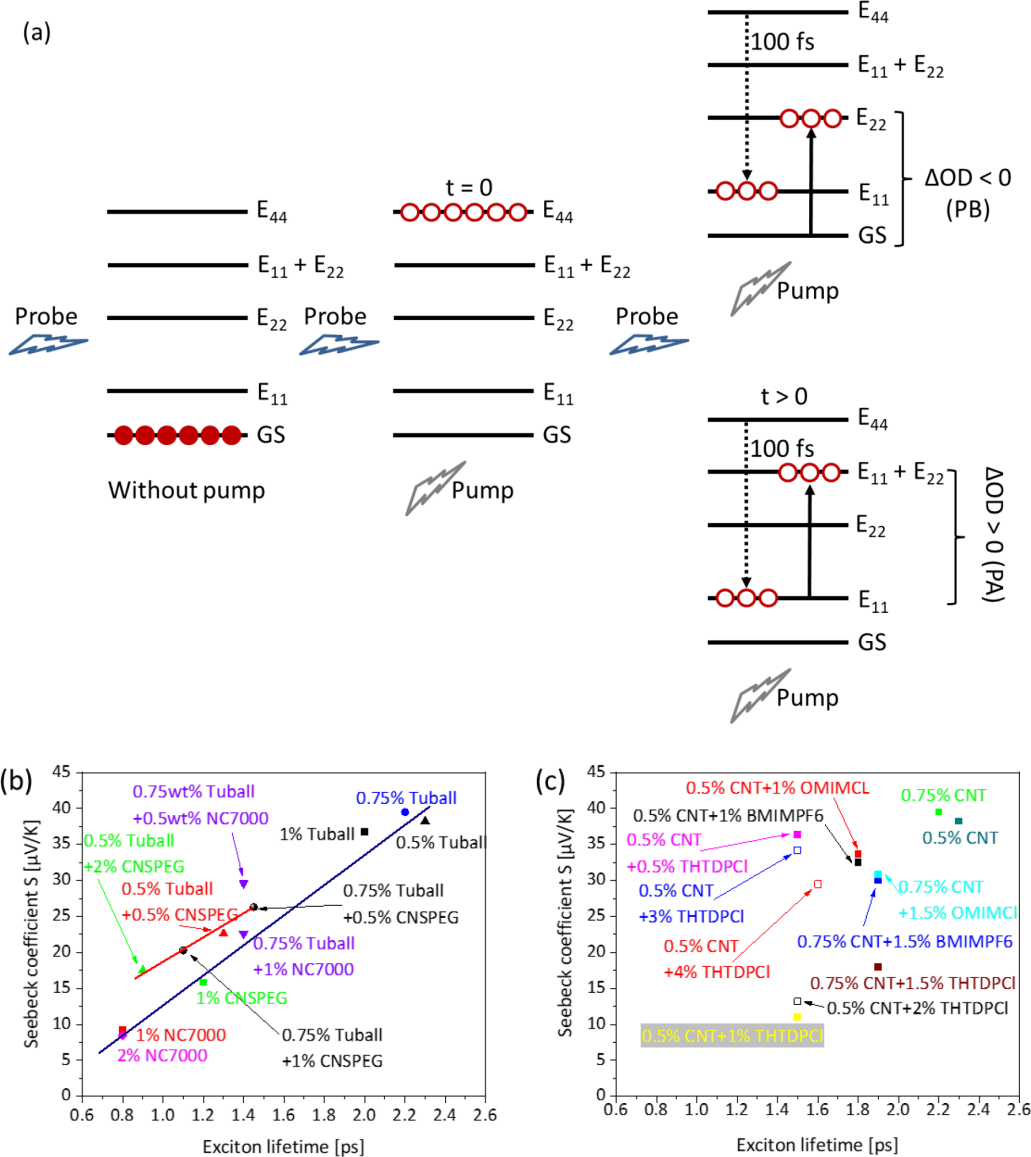
(a) Schematic representation of the relaxation processes throughout the pump-probe time-resolved transient absorption spectroscopy (TAS) experiment, without pump, with pump at *t* = 0, and with pump at *t* > 0. (b) Seebeck coefficient vs exciton lifetimes in composites without ILs, adapted from ref (33), and (c) with different ILs. The empty symbols in (c) represent negative *S* values.

Moreover, Figure 7 shows the corresponding transient carrier dynamics for the PC/0.5 wt % SWCNT + 1 wt % IL, and Figure S2 displays the PC/0.75 wt % SWCNT + 1.5 wt % IL composites, while the obtained exciton lifetimes are listed in Table S1 along with the TE parameters. The exciton lifetimes were obtained upon following typical fitting procedures that have been described explicitly in one of our previous studies, along with the detailed error analysis.^33^ It is found that apart from changing the signal of the optical density from photo-absorption to photo-bleaching, the introduction of the ILs results in a decrease in the exciton lifetimes of all composites, when compared to the IL-free PC/SWCNT samples. Indicatively, for the PC/0.5 wt % SWCNT composites, the lifetime drops from 2.3 to 1.5 ps, 1.8 ps, and 1.8 ps for THTDPCl, BMIMPF6, and OMIMCl addition, respectively (Table S1). In a similar manner, the exciton lifetimes for the PC/0.75 wt % SWCNT composites decrease from 2.2 to 1.9 ps for the three ILs (Table S1). In order to investigate the effect of ILs addition on the charge carrier characteristics in more detail, the pristine OMIMCl IL was also studied by TAS. Figure 7a depicts the transparent cuvette placed within the TAS instrument, whereas Figure 7b presents the optical density spectra at different time delays. A striking observation emerges, as it is found that the pristine OMIMCl exhibits PB optical density profiles, as was the case for the PC/0.5 wt % SWCNT and PC/0.75 wt % SWCNT composites prior to the incorporation of the ILs (Figures 7a and 8a). Also, it becomes apparent that the OMIMCl IL exhibits an ultrafast exciton lifetime of around 0.5 ps (Figure S3) when compared to those of the PC/SWCNT composites.

The aforementioned findings are rationalized as follows. In the case of PC/SWCNT composites without ILs, the obtained PB during TAS experiments arises from the rapid relaxation of excitons from higher states to the E_11_ level (within 100 fs), as depicted schematically in Figure 8a. The same situation applies for the pristine OMIMCl liquid that also exhibits negative optical density. However, the latter shows considerably faster exciton lifetimes, suggesting a considerably faster recombination of electrons with excitons. Remarkably, upon incorporating the ILs in PC/SWCNT composites, the number of excitons and excited electrons increases significantly, forcing the biexciton formation to higher energy states (E_11_ + E_22_ manifold) within the composite, as depicted schematically in Figure 8a.^57^ The latter induce the obtained PA absorption features obtained in the IL-containing composites, as less photoreceptors are located in the ground state (GS) (Figure 8a), and consequently less light is detected.

Finally, Figure 8b depicts the obtained dependence of Seebeck coefficient (*S*) as a function of exciton lifetimes for various single-filler and hybrid-filler PC composites,^33^ whereas Figure 8c shows the corresponding dependence when the various ILs are introduced in the PC/SWCNT samples (this study). A striking observation emerges from these figures. As it was discussed previously,^33^ the PC composites (in the absence of IL) exhibit an almost linear dependence of *S* with exciton lifetime (Figure 8b). The situation is quite different for the composites PC/SWCNT + IL, where the *S* is completely independent of the lifetime of the excitons (Figure 8c). The optical density profiles of the composites provide evidence on the physical mechanism of the obtained features. Indeed, for the IL-free composites, the negative optical density profiles reveal the absence of excitons and biexcitons on higher energetic manifolds. Consequently, every recombination between excitons and free electrons originates from the ground state, and thus, the exciton lifetimes probed by TAS will be dependent to the TE process. Indeed, as depicted in Figure 8b, a direct correlation between the *S* and the exciton lifetimes can be concluded. On the contrary, when the ILs are incorporated, excitons are also located in higher energetic states, as verified by the obtained PA profiles. In such a case, the relaxations attributing to the PA band, and the corresponding exciton lifetimes, also arise from inter band movements of the excitons, as well as from the recombination of excitons with free electrons. Thus, the obtained exciton lifetimes would not correlate directly to the TE processes, and the obtained dependence of *S* with exciton lifetime becomes random (Figure 8c).

In terms of electrical conductivity, the inspection of Table S1 reveals that with one exception of the PC/0.5 wt % SWCNT + 1 wt % BMIMPF6, the addition of the IL enhances the conductivity. This finding is in agreement with what was previously reported for single-filler PC composites.^33^ In particular, it was found that the electrical conductivity of single-filler PC composites depends mainly on the electron mobility within the sample, rather than the exciton lifetimes. Indeed, the electrical conductivity is based on the general equation σ = *ne*μ, where *n* is the number of free charge carriers, *e* is the electron charge, and μ is the carrier mobility. Upon introducing the ILs, the total number of charge carrier increases, and thus, the conductivity of the IL-containing composites improves when compared to the IL-free composites.

## 4 Summary

PC and PEEK were melt-mixed with SWCNT in order to generate electrically and thermoelectrically conductive composites. Positive Seebeck coefficients were measured for composites with 0.5 to 2.0 wt % SWCNTs based on both polymer matrices, thus representing p-type materials. As an additive, three different ILs were used with the goal to change the TE conduction type of the composites from p-type to n-type. It was found that in both composite types only the phosphonium-based IL was able to induce this switching. For the imidazolium-based ILs, only a reduction of the Seebeck coefficient was observed. This means that imidazolium-based ILs can change the conduction type, but in the chosen example, this property was not pronounced enough. The TE results show that the SWCNT:THTDPCl ratio plays a role on the switching efficiency. Increasing the THTDPCl amount in the PC composites first leads to a reduction of the positive *S* value from 38.2 μV/K (PC/0.5 wt % SWCNT) to 11.0 μV/K (PC/0.5 wt % SWCNT+ 1 wt % THTDPCl) and then to negative *S* values between −13.2 and −34.2 μV/K (PC/0.5 wt % SWCNT + 2–4 wt % THTDPCl). For the PEEK composites (0.5 and 0.75 wt % SWCNT), the SWCNT:THTDPCl ratio of 1:2 was already sufficient to achieve negative *S* values. In both polymer types, a plateau value of the Seebeck coefficient was found, so that increasing THTDPCl addition did not lead to more negative *S* values. For PC/SWCNT composites, the most negative *S* value was reached at −34.2 μV/K for PC/0.5 wt % Tuball + 3 wt % THTDPCl, whereas a Seebeck coefficient of −37.1 μV/K was achieved for PEEK/0.75 wt % Tuball + 3 wt % THTDPCl.

The TAS studies showed that the introduction of ILs in the developed PC/CNT composites induces excitation of excitons at higher energy states when compared to the IL-free polymer composites. As a result, the direct correlation between *S* and exciton lifetimes that was found for the IL-free composites does not hold after the incorporation of ILs. Moreover, the introduction of IL appears to reduce the exciton lifetimes evidence for faster recombination rates. Future perspectives involve the implementation of TAS studies at various temperatures and with different excitation fluences, targeting evidence for linking the signal of *S* with the optical properties of the composites, as expressed by the time-resolved optical densities.

## Supporting Information Available

Thermogravimetric analysis on ILs; TAS spectra of PC composites and IL; and thermoelectric parameters of all composites (PDF)
