# Supplementary material for: Change of Conduction Mechanism in Polymer/Single Wall Carbon Nanotube Composites upon Introduction of Ionic Liquids and Their Investigation by Transient Absorption Spectroscopy: Implication for Thermoelectric Applications
Source: ACS Appl Nano Mater. 2023 Jul 7;6(14):13027–36. doi: 10.1021/acsanm.3c01735 (PMC10391594; doi:10.1021/acsanm.3c01735)
Supplement: Supplementary file 1 — an3c01735_si_001.pdf [file an3c01735_si_001.pdf]

Supporting Information

Change of Conduction Mechanism in Polymer/Single Wall Carbon Nanotube Composites upon Introduction of Ionic Liquids and their Investigation by Transient Absorption Spectroscopy: Implication for Thermoelectric Applications

Beate Krause[*1], Ioannis Konidakis [2], Emmanuel Stratakis [2], Petra Pötschke [1]

[1] Leibniz-Institut für Polymerforschung Dresden e.V. (IPF), Hohe Str. 6, 01069 Dresden, Germany.

[2] Institute of Electronic Structure and Laser (IESL), Foundation for Research and Technology-Hellas (FORTH), 70013, Heraklion-Crete, Greece.

*Corresponding author: Krause-beate@ipfdd.de; +49 351 4658 736

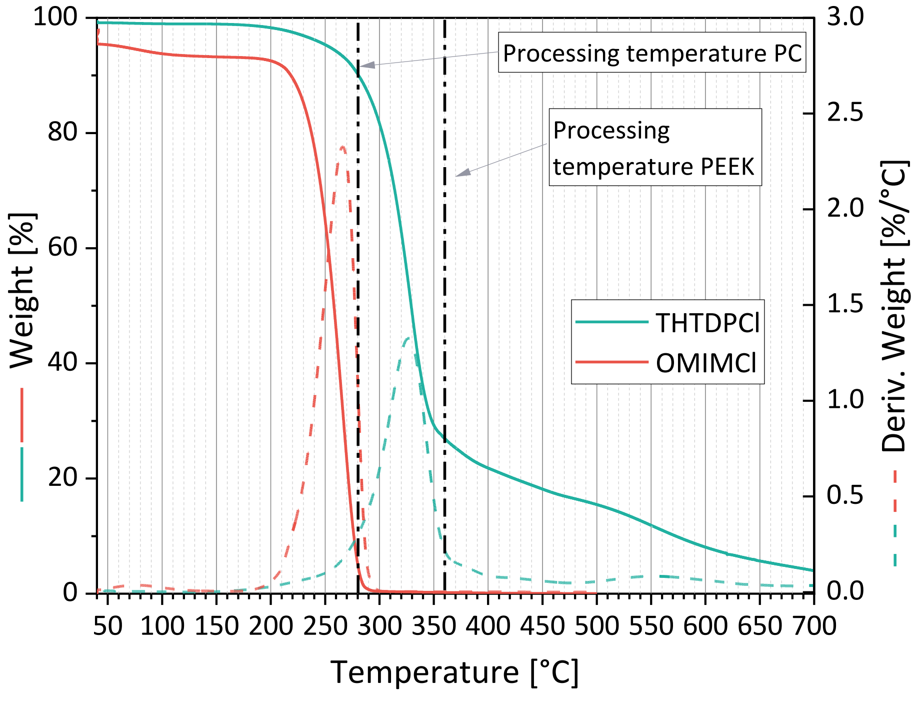

Figure S1: Thermogravimetric analysis (TGA) on ionic liquids THTDPCl and OMIMCl (TGA was performed on ionic liquids by using a Q 5000 analyzer (TA Instruments, Hüllhorst, Germany) in air atmosphere. The heating rate was 10 K/min, and a temperature range from 25 °C up to 500 or 800 °C was applied.

The maximal thermal degradation of ILs is air was determined to be at 265 °C (OMIMCl) or 325 °C (THTDPCl) (see Figure S1).

Table S1: Thermoelectric parameters and TAS results for PC/SWCNT composites

Composite	Electrical volume conductivity [S/m]	Seebeck coefficient [µV/K]	Power factor [µW/(m·K^2)]	TAS Lifetime [ps]
PC/ 0.5 wt % SWCNT [S1]	0.6	38.2 ± 0.2	8.3E-04	2.3 ± 0.1
PC/ 0.5 wt % SWCNT +1 wt % THTDPCl	0.7 ± 0.0	13.3 ± 0.2	1.2E-04 ± 8.4E-10	1.5 ± 0.1
PC/ 0.5 wt % SWCNT +1 wt % BMIMPF6	3.1 ± 0.8	32.5 ± 0.6	3.3E-03 ± 2.5E-07	1.8 ± 0.1
PC/ 0.5 wt % SWCNT +1 wt % OMIMCl	1.6 ± 0.3	33.6 ± 2.1	1.8E-03 ± 1.2E-06	1.8 ± 0.1
PC/ 0.75 wt % SWCNT [S1]	0.9	39.5 ± 0.8	1.4E-03	2.2 ± 0.1
PC/ 0.75 wt % SWCNT +1.5 wt % THTDPCl	5.7 ± 2.0	-27.6 ± 0.4	4.4E-03 ± 3.6E-07	1.9 ± 0.1
PC/ 0.75 wt % SWCNT +1.5 wt % BMIMPF6	3.7 ± 0.0	29.8 ± 0.3	3.3E-03 ± 8.0E-10	1.9 ± 0.1
PC/ 0.75 wt % SWCNT +1.5 wt % OMIMCl	3.1 ± 0.01	29.9 ± 0.4	2.7E-03 ± 1.0E-12	1.9 ± 0.1
PC/ 1 wt % SWCNT [S1]	1.0	36.7 ± 2.0	1.3E-03	
PC/ 1 wt % SWCNT +2 wt % THTDPCl	3.2 ± 0.5	-32.9 ± 0.1	3.4E-03 ± 2.8E-09	
PC/ 1 wt % SWCNT +2 wt % BMIMPF6	3.3 ± 0.1	31.1 ± 0.1	3.2E-03 ± 5.5E-10	
PC/ 1 wt % SWCNT +2 wt % OMIMCl	5.3 ± 0.1	28.5 ± 1.3	4.3E-03 ± 2.0E-07	
PC/ 2 wt % SWCNT [S1]	1.0	37.8 ± 0.3	1.2E-03	
PC/ 2 wt % SWCNT +4 wt % THTDPCl	26.4 ± 4.1	-30.5 ± 1.3	2.5E-02 ± 7.3E-06	
PC/ 2 wt % SWCNT +4 wt % BMIMPF6	13.7 ± 2.6	28.7 ± 2.7	1.1E-02 ± 1.9E-05	
PC/ 2 wt % SWCNT +4 wt % OMIMCl	17.5 ± 10.5	40.9 ± 3.0	2.9E-02 ± 9.4E-05	

Table S2. Thermoelectric parameters and TAS results for PC/0.5 wt % SWCNT composites by variation of the THTDPCl content

**Composite**	**Electrical volume conductivity [S/m]**	**Seebeck coefficient [µV/K]**	**Power factor [µW/(m·K²)]**	**TAS lifetime [ps]**
PC/ 0.5 wt % SWCNT [S1]	0.6	38.2 ± 0.2	8.3E-04	2.3 ± 0.1
PC/ 0.5 wt % SWCNT + 0.5 wt % THTDPCl	1.3 ± 0.2	36.3 ± 2.9	1.7E-03 ± 1.7E-06	1.5 ± 0.1
PC/ 0.5 wt % SWCNT +1 wt % THTDPCl	0.7 ± 0.0	13.3 ± 0.2	1.2E-04 ± 8.4E-10	1.5 ± 0.1
PC/ 0.5 wt % SWCNT +2 wt % THTDPCl	1.2 ± 0.1	-13.2 ± 1.4	2.0E-04 ± 2.0E-07	1.5 ± 0.1
PC/ 0.5 wt % SWCNT +3 wt % THTDPCl	5.1 ± 1.1	-34.2 ± 1.3	6.0E-03 ± 2.0E-06	1.5 ± 0.1
PC/ 0.5 wt % SWCNT +4 wt % THTDPCl	1.6 ± 0.1	-29.5 ± 3.7	1.3E-03 ± 1.2E-06	1.6 ± 0.1

Table S3. TE performance of PEEK/1 wt % SWCNT composites without and with 2 wt % Ionic liquids (SWCNT:IL ratio 1:2)

**Composite**	**Electrical volume conductivity [S/m]**	**Seebeck coefficient [µV/K]**	**Power factor [µW/(m·K²)]**
PEEK/1 wt % SWCNT [S1]	6.2	48.0 ± 1.3	7.2E-03
PEEK/1 wt % SWCNT + 2 wt % THTDPCl	12.5 ± 2.4	-17.4 ± 2.1	3.6E-03 ± 9.9E-06
PEEK/ 1 wt % SWCNT + 2 wt % BMIMPF6	0.7 ± 0.2	17.2 ± 4.1	2.1E-04 ± 3.0E-06
PEEK/ 1 wt % SWCNT + 2 wt % OMIMCl	0.2 ± 0.1	47.4 ± 1.1	4.9E-04 ± 8.7E-08

Table S4. TE performance of PEEK/SWCNT composites (0.5-0.75 wt % SWCNT) without and with different amounts of Ionic liquids

**Composite**	**Electrical volume conductivity [S/m]**	**Seebeck coefficient [µV/K]**	**Power factor [µW/(m·K^2)]**
PEEK/0.5 wt % SWCNT [S1]	2.2	59.4 ± 1.2	7.6E-03
PEEK/0.5 wt % SWCNT + 1 wt % THTDPCl	0.4 ± 0.1	-7.0 ± 0.3	2.0E-05 ± 7.3E-09
PEEK/0.5 wt % SWCNT + 2 wt % THTDPCl	1.7 ± 0.5	-28.4 ± 2.0	1.3E-03 ± 1.9E-06
PEEK/0.5 wt % SWCNT + 3 wt % THTDPCl	2.7 ± 0.6	-29.8 ± 1.16	2.4E-03 ± 8.4E-07
PEEK/0.75 wt % SWCNT [S1]	1.8	61.3 ± 0.2	6.6E-03
PEEK/0.75 wt % SWCNT + 1.5 wt % THTDPCl	5.7 ± 2.0	-27.6 ± 0.4	3.8E-03 ± 3.6E-07
PEEK/0.75 wt % SWCNT + 2 wt % THTDPCl	1.7 ± 0.2	-30.0 ± 0.7	1.5E-03 ± 8.2E-08
PEEK/0.75 wt % SWCNT + 3 wt % THTDPCl	7.1 ± 0.6	-37.1 ± 0.2	9.8E-03 ± 2.4E-08

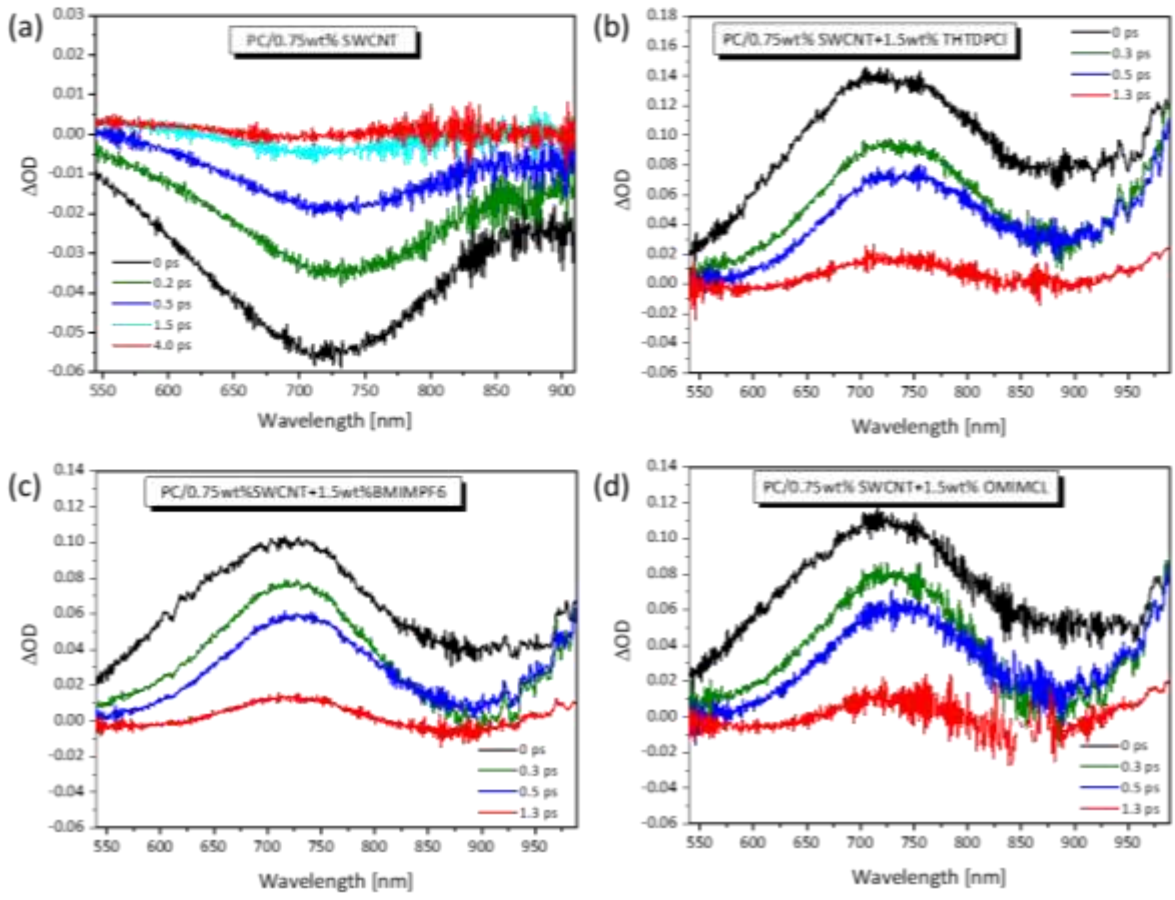

Figure S2. TAS spectra of the difference in optical density (ΔOD) as a function of wavelength for various delay times of (a) PC/ 0.75 wt % SWCNT, and PC/ 0.75 wt % SWCNT + 1.5 wt % IL, where the used IL is (b) THTDPCl, (c) BMIMPF6, and (d) OMIMCL.

(a) 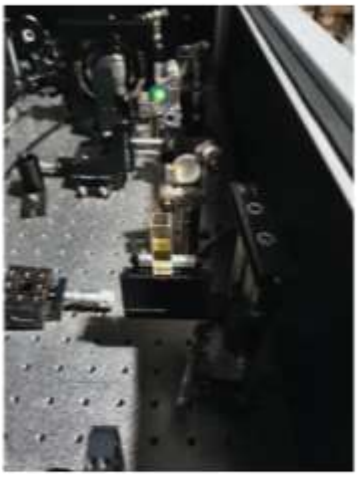 (b) 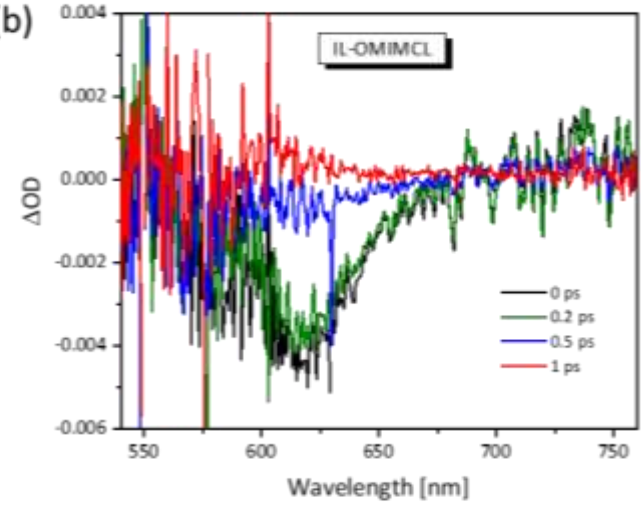

Figure S3. (a) OMIMCl IL inside cuvette within the TAS setup, (b) TAS spectra of the difference in optical density (ΔOD) as a function of wavelength for various delay times of OMIMCl IL.

References:

[S1] Konidakis, I; Krause, B.; Park, G. H.; Pulumati, N.; Reith, H.; Pötschke, P.; Stratakis, E. Probing the carrier dynamics of polymer composites with single and hybrid carbon nanotube fillers for improved thermoelectric performance. ACS Appl. Energy Mater. **2022**, *5*, 9770.
